# CD103-Negative Hairy Cell Leukemia: A Case Report From University Teaching Hospital, Zambia

**DOI:** 10.1155/carm/7084757

**Published:** 2025-07-11

**Authors:** Natasha Mupeta Kaweme, Sahar Mounir Nagib Butress, Inonge Akekelwa, Sumbukeni Francis Kowa, Hamakwa Muluti Mantina, Charles Kapela Mwandama

**Affiliations:** ^1^Department of Pathology and Microbiology, Adult Hospital, University Teaching Hospital, Haematology Unit, Private Bag RW 1X, Lusaka, Zambia; ^2^Department of Pathology and Microbiology, Adult Hospital, University Teaching Hospital, Private Bag RW 1X, Lusaka, Zambia; ^3^Department of Internal Medicine, Adult Hospital, University Teaching Hospital, Haematology and Oncology Unit, Private Bag RW 1X, Lusaka, Zambia

**Keywords:** BRAF V600E, cladribine, clinical presentation, hairy cell leukemia, immunophenotype

## Abstract

Hairy cell leukemia (HCL) is among a group of mature lymphoid B-cell disorders characterized by the identification of hairy cells and a unique genetic profile. Detection of CD103 expression on flow cytometry is the key in enumerating the immunologic score for diagnosing HCL. For a disease that is more prevalent in Caucasians and less common in African populations, we report an unusual case of CD103-negative classical HCL in a 43-year-old African male, who presented with refractory anemia, thrombocytopenia, and splenomegaly. In patients with refractory anemia, it is crucial to consider investigating HCL, as this may influence therapeutic decisions and, consequently, overall patient outcomes.

## 1. Introduction

Hairy cell leukemia (HCL) is a rare, chronic malignancy of mature B lymphocytes, accounting for less than 2% of all leukemia. It exhibits a male predominance, occurring four to five times more frequently in men than in women. The cause of this disparity is unexplained [1]. In the United States, the incidence of classic HCL (HCLc) and hairy cell leukemia-variant (HCLv) was 0.3 per 100,00 and 0.2 per 100,000, respectively [2]. There is a paucity of information on the prevalence and incidence of HCLc in Africa and Zambia, in particular. From the literature search, this may represent the first documented case of HCLc reported in Zambia.

Few reports have described HCLc in sub-Saharan Africa. A retrospective study conducted between 1993 and 1999 in Brazzaville, Congo, identified 10 cases of HCL with pancytopenia and splenomegaly although their symptoms were initially misattributed to malaria [3]. In the Democratic Republic of Congo, a highly unusual case of HCL was reported in a 4-year-old child, emphasizing its rarity in African and pediatric populations [4]. Furthermore, in North Africa, Tlamcani et al. reported three cases diagnosed at CHU Hassan II in Fès, Morocco, between 2015 and 2017, highlighting the importance of advanced diagnostics in identifying HCL in low-resource settings [5].

HCLc affects Caucasians more than African Americans and is most commonly reported in middle-aged to older males, with infrequent occurrences in children [6]. The median age at diagnosis is approximately 63 years in males and 59 years in females [1]. While the etiology of HCLc remains largely unknown, suggested risk factors include exposure to pesticides, petroleum products, and ionizing radiation [7].

The pathogenesis of HCLc is driven by the *B-raf proto-oncogene (BRAF) V600E* mutation [8]. Inactivation of *CDKN1B* occurs in 16% of the patients, making it the second most common mutated gene in HCLc [9]. HCLv often harbors activating mutations in *MAP2K1,* the gene encoding MEK1, with a reported prevalence of circa 30% [10]. First-line treatment in HCLc includes cladribine or pentostatin [11, 12]. Rituximab enhances the clearance of minimal residue disease [13]. In relapsed or refractory cases, *BRAF* inhibitors [14, 15], with or without MEK inhibitors [16], and Bruton's tyrosine kinase inhibitors are emerging options [17, 18].

We report a confirmed case of CD103-negative HCLc in Zambia, diagnosed at the University Teaching Hospital. This case underscores the need to bring attention to this rare leukemia for prompt diagnosis and effective treatment.

## 2. Case Presentation

A 43-year-old male presented in April 2024 with a 2-month history of generalized body weakness, fatigue, and cough. He reported prior pneumonia and refractory anemia during this period. Initial investigations revealed anemia and thrombocytopenia, prompting hematology consultation. On examination, he was mildly pale with splenomegaly and no lymphadenopathy. Abdominal ultrasound confirmed splenomegaly measuring 15 cm below the costal margin.

Evaluation of laboratory findings showed a normal white cell count with neutropenia, moderate anemia, and thrombocytopenia. Monocytopenia was absent in the differential count. Other investigations did not reveal any significant abnormalities. The preliminary investigation results are presented in Table 1.

**Figure 1:**
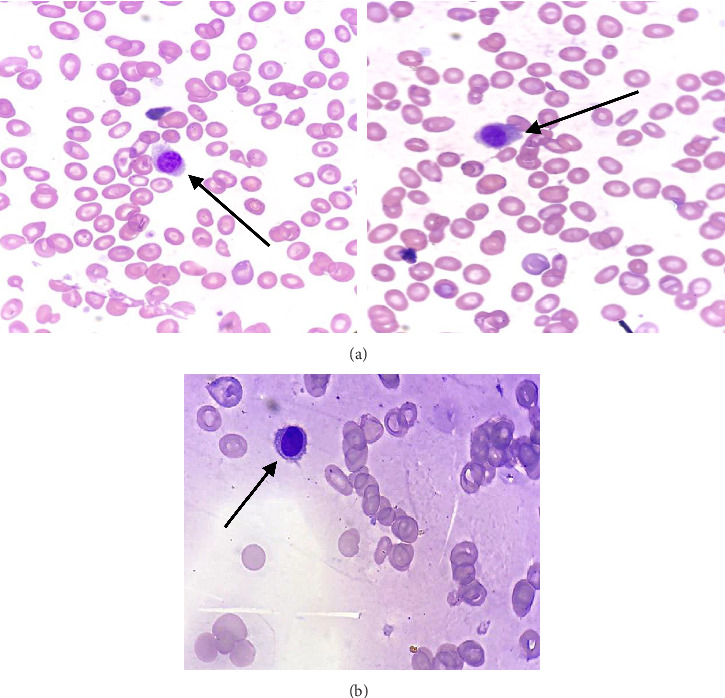
The morphology of hairy cells in the peripheral blood (a) and the bone marrow (b) (Wright–Giemsa, ×100).

Peripheral blood film showed anisocytosis, target cells, polychromasia on red blood cells, atypical lymphocytes with cytoplasmic “hairy” projections, and thrombocytopenia. Bone marrow aspirates performed yielded “dry taps.” Nonetheless, a small number of lymphocytes with cytoplasmic projections were observed (Figure 1).

**Figure 2:**
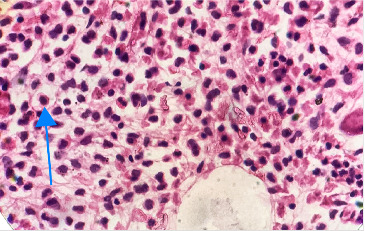
Bone marrow biopsy showing atypical lymphoid cells with abundant pale cytoplasm and clear cytoplasmic borders, giving the classic “fried egg” appearance (Hematoxylin and eosin, ×100).

In light of the dry tap results and the distinct lymphocyte morphology, a differential diagnosis of HCL was considered. Bone marrow trephine biopsy revealed a hypercellular marrow with diffuse infiltration of atypical lymphoid cells displaying round nuclei and abundant pale cytoplasm, giving the cells a “fried egg” appearance (Figure 2). The marrow background was hypocellular for normal trilineage hematopoiesis. These morphological features raised suspicion for HCLc infiltration, which was subsequently confirmed by flow cytometry.

Multiparameter flow cytometry performed on peripheral blood using BD FACS CANTO II/BD FACS DIVA, 8-Colour flow cytometry showed a lymphocyte population of 74.0% with a positive expression for CD19, CD20, CD200, CD10, CD38, and FMC7 and negative expression for CD5, CD23, Kappa, and CD103. Both CD11c and CD25 were positive. Our patient's immunological score was 2 based on positive CD11c and CD25. However, CD123 was not tested.

Mutation analysis of the *BRAF* gene using polymerase chain reaction (PCR)-Sequencing detected a *BRAF V600E* mutation (NM_ 004333.4: c.1799T > A (p. Val600Glu). Given the clinical findings of neutropenia, anemia, thrombocytopenia, and splenomegaly, coupled with the presence of hairy cells in blood film and bone marrow, as well as positivity for CD11c, CD25, and the BRAF mutation, the patient was diagnosed with HCLc.

Cladribine was prescribed at a dosage of 0.1 mg/kg/day for 7 days. However, treatment only commenced on July 9, 2024, 25 days later, due to the unavailability of the drug locally. Blood counts began to recover after a brief decline at the beginning of treatment. Blood routine on Day 17 after treatment initiation showed pancytopenia with Hb 8.7 g/dL, PLT 43 × 10^9^/L, and ANC 0.10 × 10^9^/L, but the patient remained stable and asymptomatic. By Day 34, blood counts improved significantly with Hb 11.6 g/dL, PLT 243 × 10^9^/L, and ANC 3.38 × 10^9^/L, and the patient was discharged. Subsequent reviews showed normalization of PLT and ANC counts, although mild anemia persisted. Currently, the patient remains in hematologic remission and reports good health.

## 3. Discussion

We discuss a male patient diagnosed with HCLc based on immunophenotyping and molecular analysis. Clinical features included refractory anemia, thrombocytopenia, and splenomegaly caused by the infiltration of malignant hairy cells in the bone marrow and spleen. Notably, monocytopenia was absent, an uncommon finding in HCLc. We hypothesize this may be due to insufficient marrow infiltration in early HCLc or a reactive process due to inflammation. Morphology revealed “hairy looking” cells on cytology and the characteristic “fried egg” appearance on H&E staining. Immunophenotyping demonstrated CD11c, CD25, and CD10 positivity. Noteworthy is that the patient showed an atypical immunophenotype, CD103, negativity, a rare appearance in HCLc. Given the nonspecificity of immunophenotyping in isolation, molecular detection of the *BRAF V600E* mutation was valuable in achieving an accurate diagnosis.

Zhao and colleagues reported a similar case of a patient who presented with fatigue, pancytopenia with monocytopenia, and splenomegaly and was diagnosed with CD103-negative and CD23-positive HCLc, alongside a positive *BRAF V600E* mutation [13]. While CD103-negativity is uncommon in HCLc, the incidence of CD23 positivity ranges from 17% to 62.5% [13, 19]. In contrast, Jimenez and Shuai described a rare and diagnostically challenging *BRAF*-negative/CD103-negative case of HCLc. Clinically, the patient demonstrated typical features, including neutropenia, thrombocytopenia, and splenomegaly. Molecular testing for Annexin A1 confirmed the diagnosis [20]. Although our case did not necessitate Annexin A1 staining, we recognize its importance in atypical presentations. Chen et al. described CD103-/CD10+ and CD10+/CD23+ HCL immunophenotypic variations and found no significant differences in clinical presentation compared with HCLc [21].

HCLc cells are generally negative for CD5, CD10, and CD23 [21]. However, our patient showed positive CD10 expression, which is associated with lymphoid cells of follicular center origin. CD10 positivity can also be found in other B-cell disorders, such as precursor B-cell acute lymphoblastic leukemia and germinal center-related diffuse large B-cell lymphomas [22]. Although CD10 positivity in HCLc is rare, occurring in 5%–26% of the cases [21], it may lead to misdiagnosis as follicular lymphoma, known for coexpressing CD10, CD20, and BCL6. Consequently, additional testing with *BRAF* mutation, FISH for *t* [14, 18], and Annexin A1 may aid in accurate differential diagnosis. Jasionowski et al. demonstrated that about 10% of HCLc cases exhibited aberrant CD10 expression while sharing morphological and clinical similarities with CD10-negative HCL, highlighting its importance in evaluating specimens with limited morphologic and immunophenotype characteristics [23].

Additional immunophenotypic aberrancies, such as CD5 or CD38 positivity [24], are less common but associated with poorer prognosis [25]. Typically, laboratory findings show a positive *BRAF* mutation and the absence of *t* [11, 14] in CD5-positive HCLc. CD5 positivity necessitates the exclusion of CLL and MCL [19, 26–28]. CD38 expression occurs in 14%–30% of HCLc cases, and because of its association with poor prognosis, it is investigated for CD38-targeted therapy [25]. CD123-negativity has also been reported although its clinical significance remains unclear [29].

Differentiating HCLc from other B-cell disorders, such as splenic marginal zone lymphoma (SMZL), splenic B-cell lymphoma/leukaemia with prominent nucleoli (SLPN), encompassing HCLv and CD5 negative B-prolymphocytic leukemia (B-PLL), and splenic diffuse red pulp small B-cell lymphoma (SDRPL) is critical [30]. HCLc typically presents with pancytopenia, splenomegaly, and marrow fibrosis [1, 31], while HCLv often shows leukocytosis and follows a more aggressive clinical course. Both SMZL and SDRPL may also present with splenomegaly [32]. Morphologically, HCLc cells exhibit characteristic “hairy” projections, round or kidney-shaped nuclei, and inconspicuous nucleoli. In contrast, HCLv cells have fewer hair-like projections and more prominent nucleoli. SMZL and SDRPL cells have long, nonpolar villi compared with HCLc cells [32].

On immunophenotyping, HCLc expresses CD11c, CD25, CD103, and CD123, which define the immunologic score of HCLc. A score of ≥ 3 points is observed in 98% of the HCLc cases [31]. In HCLv, CD25 is negative in all cases, with dim or negative expression of CD103 and CD123. SMZL and SDRPL are typically negative for CD103 and CD123 [19, 32]. Molecular detection of the *BRAF V600E* mutation is found in ≥ 95% of the HCLc cases [33] but is absent in HCLv, SMZL, and SDRPL [32]. Annexin A1 is specifically expressed in HCLc and not in the other entities [19]. Differentiating HCLc from other HCL-like disorders is crucial for treatment, as purine nucleoside analogs induce long-term complete remission in 85%–90% of the HCLc cases [34, 35], while only 50% of HCLv patients achieve partial remission [36].

Our patient exhibited adverse prognostic markers, including splenomegaly, circulating hairy cells, and CD38 expression, although LDH was normal. Treatment is indicated in patients with cytopenias (Hb < 11 g/dL; PLT < 100 × 10^9^/L; and ANC < 1.0 × 10^9^/L) or symptomatic splenomegaly [11]. The patient met all criteria and showed excellent response to cladribine. This is despite the fact that he is male and black African, all associated with poor prognosis. Cladribine or pentostatin is not readily available at our facility, which can delay timely treatment initiation.

## 4. Conclusion

HCLc is among a group of mature lymphoid B-cell disorders characterized by hairy cells and a unique genetic profile. Accurate diagnosis relies on careful morphologic evaluation, immunophenotyping, and detection of the *BRAF* mutation. Limited access to advanced diagnostic techniques creates a confounding challenge with diagnosing HCLc in patients with misleading presentations such as refractory anemia and lack of visible hairy cells on blood film. In this case, although a few atypical lymphocytes were observed on the blood film, HCLc was not initially considered due to the patient's age and short duration of illness. A trephine biopsy then became crucial to confirm marrow infiltration. Given the rarity of HCLc in individuals of African descent, this case underscores the importance of considering this diagnosis as it can influence treatment decisions and patient outcomes. With limited data on the prevalence of HCLc in Africa, we strongly recommend further studies to better define the prevalence, immunophenotype, and genetic landscape of HCLc in this population.

## Figures and Tables

**Table 1:** Preliminary laboratory results.

Test	Result	Normal range
Complete blood count		
Hemoglobin (Hb) (g/dL)	8.1	14.3–18.3
White cell count (WCC) (×10^9^/L)	7.45	4.0–10.0
Platelet count (PLT) (×10^9^/L)	54	150–400
Manual differential count		
Neutrophils (*N*) (%)	6.5%	60%–70%
Absolute neutrophil count (ANC) (×10^9^/L)	0.48	2.0–7.0
Lymphocytes (*L*) (%)	40.4%	30%–40%
Monocytes (*M*) (%)	52.5%	2%–6%
Biochemistry		
Lactate dehydrogenase (LDH) (U/L)	262	230–460
C-reactive protein (CRP) (mg/L)	4.93	0–6
Erythrocyte sedimentation rate (ESR) (mm/hr)	56	0–14

## Data Availability

The original contributions presented in the study are included within the article. Further inquiries can be directed to the corresponding author.

## Nomenclature

ANC: Absolute neutrophil count
CBC: Complete blood count
HCLc: Classic hairy cell leukemia
HCLv: Hairy cell leukemia-variant
LDH: Lactate dehydrogenase
